# Hydrocarbon potential in the Northern Egyptian Red Sea: insights from geophysical datasets and analysis of onshore marginal outcrop analogues and subsurface sequences

**DOI:** 10.1038/s41598-024-79605-7

**Published:** 2025-01-07

**Authors:** Ahmed M. Noureldin, Mustafa Hassan, Mohamed Farouk, Walid M. Mabrouk, Ahmed E. Radwan, Ahmed Metwally

**Affiliations:** 1 Geophysics Department, Faculty of Science, Cairo University, Giza, 12613 Egypt; 2 Faculty of Geography and Geology, Institute of Geological Sciences, Jagiellonian University, Gronostajowa 3a, 30-387, Krakow, Poland; 3 General Petroleum Company, Nasr City, Egypt; 4 Ganoub El Wadi Petroleum Holding Company, Nasr City, Egypt

**Keywords:** Northern Egyptian Red Sea, Gebel Duwi, Southern Gulf of Suez, Hydrocarbon potential, Geology, Geophysics, Sedimentology, Seismology

## Abstract

The Red Sea remains a largely under-explored basin, with the Northern Egyptian Red Sea requiring further investigation due to limited borehole data, sparse case studies, and poor seismic quality. A petroleum system, regional structural cross-section, and geological block diagrams integrating onshore fieldwork from Gebel Duwi and offshore subsurface geology were utilized to assess the hydrocarbon potential of the Northern Egyptian Red Sea (NERS). The findings highlight that pre- and syn-rift organic-rich source units in the NERS could generate oil and gas, similar to the capped reservoirs of the Southern Gulf of Suez. The study also reveals that both regions (NERS and SGOS) were influenced by the Levant-Aqaba transform fault influenced both regions (NERS and SGOS), resulting in southwest-dipping strata, replicating the Northern Gulf of Suez structural style. Additionally, rifting extended the rotated basement faulted blocks from the shoreline to the axial trough, creating wedged Miocene sections. Most offshore boreholes in the Red Sea bottomed in Precambrian rock directly below the syn-rift Miocene sequence, suggesting these wells were off-structure, similar to sidetracked wells in the Gulf of Suez, which successfully captured thick pre- and syn-rift sediments. These insights can drive further advancements in hydrocarbon exploration in the Northern Egyptian Red Sea.

## Introduction

The Red Sea is a grossly under-explored basin in all the countries that share the Red Sea including Egypt^25,3^, Saudi Arabia^7^, Yemen, Eritrea^7^, and Sudanese Red Sea^30^ basins/sections. Previous investigations have been conducted to better understand the complexities of prospective hydrocarbon deposits^34^. However, the exploration opportunities still need to be clarified and require further deliberations^17^ and ^39^. Despite the discouraging discoveries of hydrocarbon reserves, thus far, the Red Sea still harbors undiscovered hydrocarbon potential, as most drilled wells exhibit typical signs of hydrocarbons^20^.

**Fig. 1:**
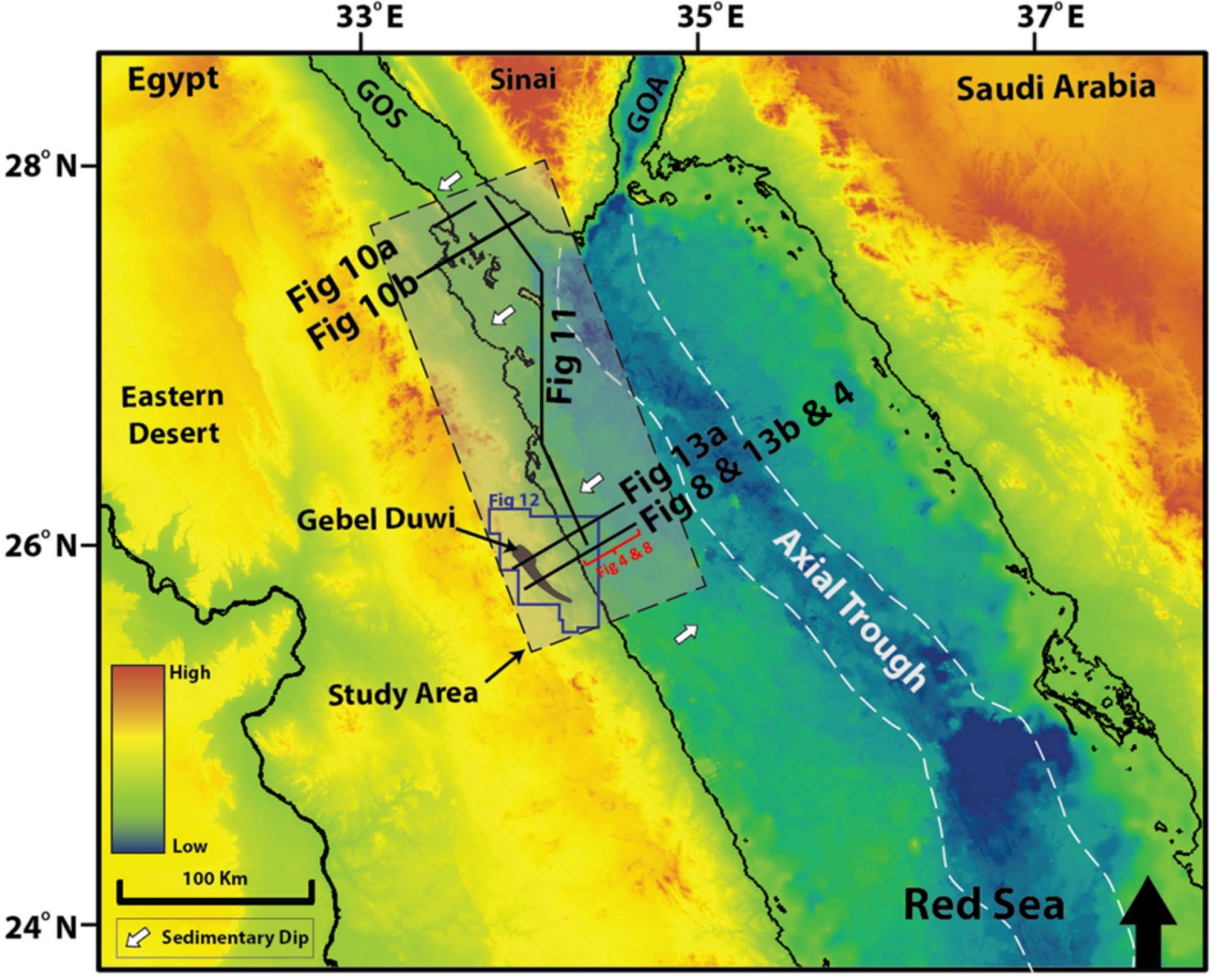
The Northern Red Sea topography map showing the location of the study area. Figure 11 links the offshore Southern Gulf of Suez and the offshore North Egyptian Red Sea, the location of Gebel Duwi is highlighted, Figs. 10a and b are referenced surface-to-subsurface dip-oriented geological cross-sections modified after^16^, and Fig. 13a, and 13b are newly surface-to-subsurface dip-oriented geo-seismic cross-sections/schemes/block diagrams, the red dashed rectangle delineates the research area of interest, Fig. 8 represents the offshore side of Figure 13b. Figure 12 is outlined over the Duwi area. GOA is the Gulf of Aqaba, and GOS is the Gulf of Suez^26^. GEBCO’s 2023 model is used for the geographical projection.

The exploration activities encounter various challenges in the Red Sea region and the North Egyptian Red Sea (Fig. 1) in particular, such as navigating the deep-water column, addressing the complexities associated with Miocene mobile salt deposits, mitigating the effects of intricate rotational block faulting, managing abnormally high pressures and very high temperatures, and overcoming limitations posed by poor subsurface seismic imaging. Gordon et al.^28^ studied the hydrocarbon potential of the North Egyptian Red Sea, concluding that key elements of the Gulf of Suez petroleum system exist in the North Egyptian Red Sea ([e.g., ^4^ and ^49^). Source-to-oil correlations in the Northern Egyptian Red Sea demonstrate that massive reservoir-limited structural fault blocks are actively charged by pre-rift^16^ and Mesozoic source rock^8^. Despite the erosion observed in Nubian facies on the crests of rotational basement fault blocks, seismic mapping indicates that the scale of the fault block in the Northern Egyptian Red Sea is similar to that in the Gulf of Suez, where pre-rift reservoirs are currently in production (e.g., ^29^ and ^57^).

Recent volcanism in the Northern Red Sea plays a significant role and should be highlighted in the discussion. Cochran^20^ examines the nucleation of an oceanic spreading center within the continental rift of the Northern Red Sea, providing foundational insights into the region’s volcanic activity. Cochran and Karner^78^ discuss the transition from rifting to drifting in the Red Sea, emphasizing the deformation and rupturing of the continental lithosphere and its impact on volcanic processes. Ali et al.^79^ present geophysical evidence for magmatism southwest of the Brothers Islands, offshore Quseir, Egypt, which sheds light on recent volcanic activity in this area. Further, Ali et al.^80^ use reflection seismic and 3D inversion of gravity and magnetic data to image Pleistocene volcanic edifices along the Egyptian Red Sea margin, offering detailed insights into volcanic structures. Ehrhardt and Hübscher^81^ review the transition from rifting to drifting in the northern Red Sea, providing lessons learned from deep ocean studies that are relevant to understanding the region’s volcanic evolution.

Building upon previous investigations within this original research article, an extensive examination of the petroleum system in the Northern Egyptian Red Sea and the conceptual framework of the onshore Egyptian Red Sea and the southern Gulf of Suez has been systematically discussed. Furthermore, an onshore-offshore correlation was established to delineate the nascent basin’s relationship with proximate analogs, facilitating a comprehensive comprehension of the petroleum potential within the examined region.

This article aims to study the petroleum system, the regional structural concept, and the transitional style between outcrops, the Southern Gulf of Suez, and the Egyptian Red Sea subsurface to unlock the hydrocarbon potential of the North Egyptian Red Sea. It is worth mentioning that most of the information about the Gulf of Suez or other nearby regions was primarily intended to serve as an analog for assessing the hydrocarbon potential in the Northern Egyptian Red Sea.

## Tectonostratigraphic settings and hydrocarbon exploration history in the Red Sea

### Cambrian to eocene: passive margin development

**Fig. 2:**
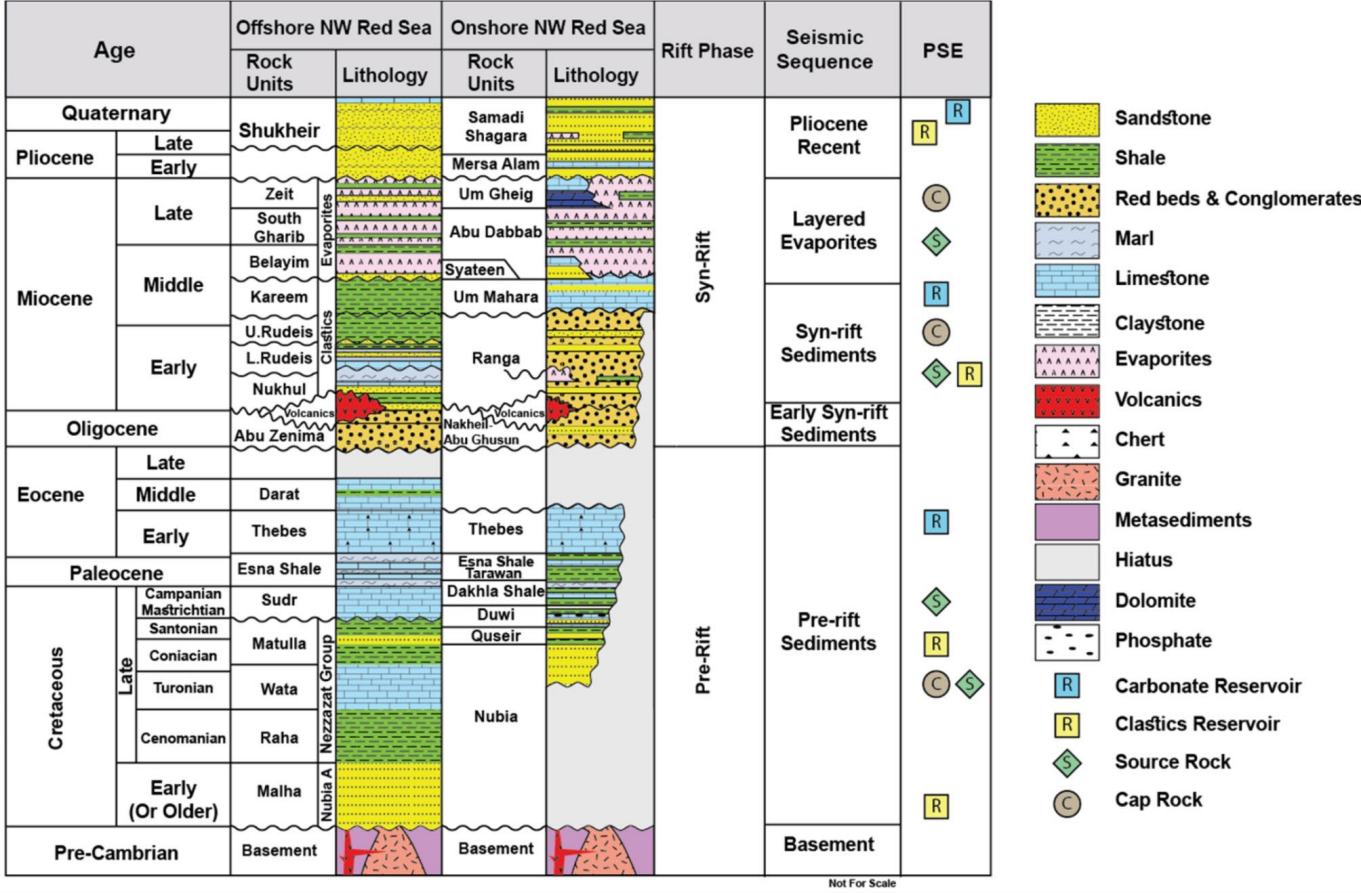
The northern Red Sea’s onshore and offshore stratigraphic column modified after^19^, (PSE: Petroleum System Elements).

The earliest sedimentary layers in the Northern Red Sea region cover the Cambrian to the Eocene (Fig. 2), depicting a passive margin development. From the Cambrian to the Early Cretaceous, the prevailing conditions were continental, leading to the accumulation of a substantial siliciclastic layer along the Neo-Tethyan margin, containing high-quality sandstones of the Nubian Group^5,6,15,63^. Subsequently, a significant transgression occurred in the Cenomanian, depositing a mixed carbonate and siliciclastic sequence from the Late Cretaceous to the Eocene, which includes crucial source rock intervals such as the Duwi and Dakhla formations^8^.

### Oligocene: Continental conditions

At the onset of the Oligocene, a global sea level decrease led to a return to continental conditions, resulting in a regional unconformity above the primary pre-rift succession^4^. A continental sequence formed across the central-northern Red Sea and Gulf of Suez called the Abu Zenima (Tayiba) Formation (Fig. 2). During the Oligocene volcanic activity related to plumes started in the Afar region and southwest Yemen, spreading northward into western Saudi Arabia^15^.

### Early miocene: occurrence of rift

Continental rifting commenced in the Gulf of Aden and the southernmost Red Sea^15^. Then, synchronous orthogonal rifting was initiated along the entire length of the Red Sea and the Gulf of Suez^15^. A marine connection with the Neo-Tethys formed to the north, leading to the deposition of a fully marine, mixed siliciclastic–carbonate sequence with potential source rocks and reservoirs^63,16,8,1^. The initiation of rifts was marked by short-lived minor basaltic volcanism and the intrusion of rift-parallel dykes, primarily in the northeast Red Sea margin^33,46,70^.

The earliest rift sedimentation transitioned from continental to shallow-marine facies (Nukhul Formation) (Fig. 2), followed by a deepening marked by organic-rich marl, shale, and sandstone within rifted lows and limestones over outboard highs (Rudeis Formation)^16,21^.

### Middle miocene: rift development and sedimentation

According to^33,21^ during the middle Miocene, the rift’s shoulders started to rise relatively, and coarse sands and conglomerates were deposited^8,6^ around its borders. In the Middle Miocene, the basin shallowed, characterized by a mixed siliciclastic and evaporite sequence (Kareem Formation) (Fig. 2).

### Late miocene: Dead Sea-aqaba transform fault and structural changes

The African plate shifted eastward to northward in transitioning from the Lower Cretaceous to the Santonian period. This change in motion led to a compressional event affecting the entire African tectonic plate^24,42,64,50,51,49,11,13,11,93,94,96^. Phases of left-lateral shear along this fault system, which currently stretches from the Red Sea rift in the Aqaba Gulf to the Maras Triple Junction in Southeast Turkey, may have had an impact on the Offshore Northern Red Sea area structural style^6^. The system has a discernible post-Pliocene pulse today and has been active since the Lower-Upper Miocene^57,58,27,15,6,53^. The Egyptian offshore fault system completely crosses the Upper Miocene and Pliocene in the Hurghada offshore region. This most likely has something to do with the recent Levant-Aqaba transform fault in the North Egyptian Red Sea^16^.

Bosworth and Burke^15^ illustrated the collision of Eurasia with Arabia caused sinistral strike-slip motion along the Dead Sea-Aqaba transform fault^21^. This structural isolation of the Gulf of Suez from the rest of the Red Sea Rift terminated rifting in the Gulf of Suez^33^.

### Pliocene to recent: oceanic accretion and modern conditions

Crustal separation and the initiation of oceanic accretion in the southern Red Sea began and progressed northward into the central and northern Red Sea^15^. By the Early Pliocene, the Red Sea connected with the Indian Ocean, reinstating fully marine conditions^16^. A Pliocene–Recent sequence developed over the Late Miocene salt, comprising a lower siliciclastic unit and an upper carbonate unit^33^.

The nature of the crust in the Northern Red Sea remains debated, with several perspectives offered. Bonatti^83^ describes the punctiform initiation of seafloor spreading during the transition from continental to oceanic rifting. Ali et al.^84^ provide constraints on Red Sea rifting in central Egypt, enhancing our understanding of the crustal characteristics. Ligi et al.^85^ attribute the initial burst of oceanic crust accretion to edge-driven mantle convection, while their later work^86^ suggests oceanic-type basaltic melt intrusions preceded continental rupture. Gaulier et al.^87^ offer seismic data on the crustal structure, and Augustin et al.^88^ review 13 million years of seafloor spreading in the Red Sea Basin, contributing to the discussion on crustal evolution.

Contourite deposits within the Plio-Pleistocene sequence are significant for understanding past oceanographic conditions and sedimentary processes. Mitchell et al.^82^ highlight how contourite-like deposits in the Plio-Pleistocene Red Sea indicate stronger-than-present circulation patterns, offering insights into historical oceanographic conditions and sedimentary dynamics.

Salt mobilization has led to the formation of salt anticlines, diapirs, and rim-synclinal basins, creating potential structural traps^15^. Understanding salt diapirs and their mobilization is crucial for predicting hydrocarbon traps in the Red Sea. Ali et al.^73^ provide insights into Middle to Late Miocene salt tectonics in the central Egyptian Red Sea, while Mitchell et al.^74^ explore submarine salt flows and their impact on salt diapirs. Mitchell et al.^75^ further, discuss the topography and deformation of Miocene salt deposits, while Smith and Santamarina^76^ review Red Sea evaporites’ formation, creep, and dissolution, highlighting their role in forming geological traps.

### Exploration activities insights across the whole Red Sea

To provide a comprehensive understanding of our study area in Egypt, it is crucial to recognize the coherent burial history and petroleum systems along the Red Sea, despite regional variations. This coherence can be attributed to the shared sequence stratigraphy^67^, which reflects common geological processes that have shaped the region’s stratigraphy^67,33^. Understanding the sequence stratigraphy is fundamental for effective petroleum exploration and resource assessment, as it offers a framework for predicting the distribution and characteristics of hydrocarbon-bearing formations^32,41^.

**Fig. 3:**
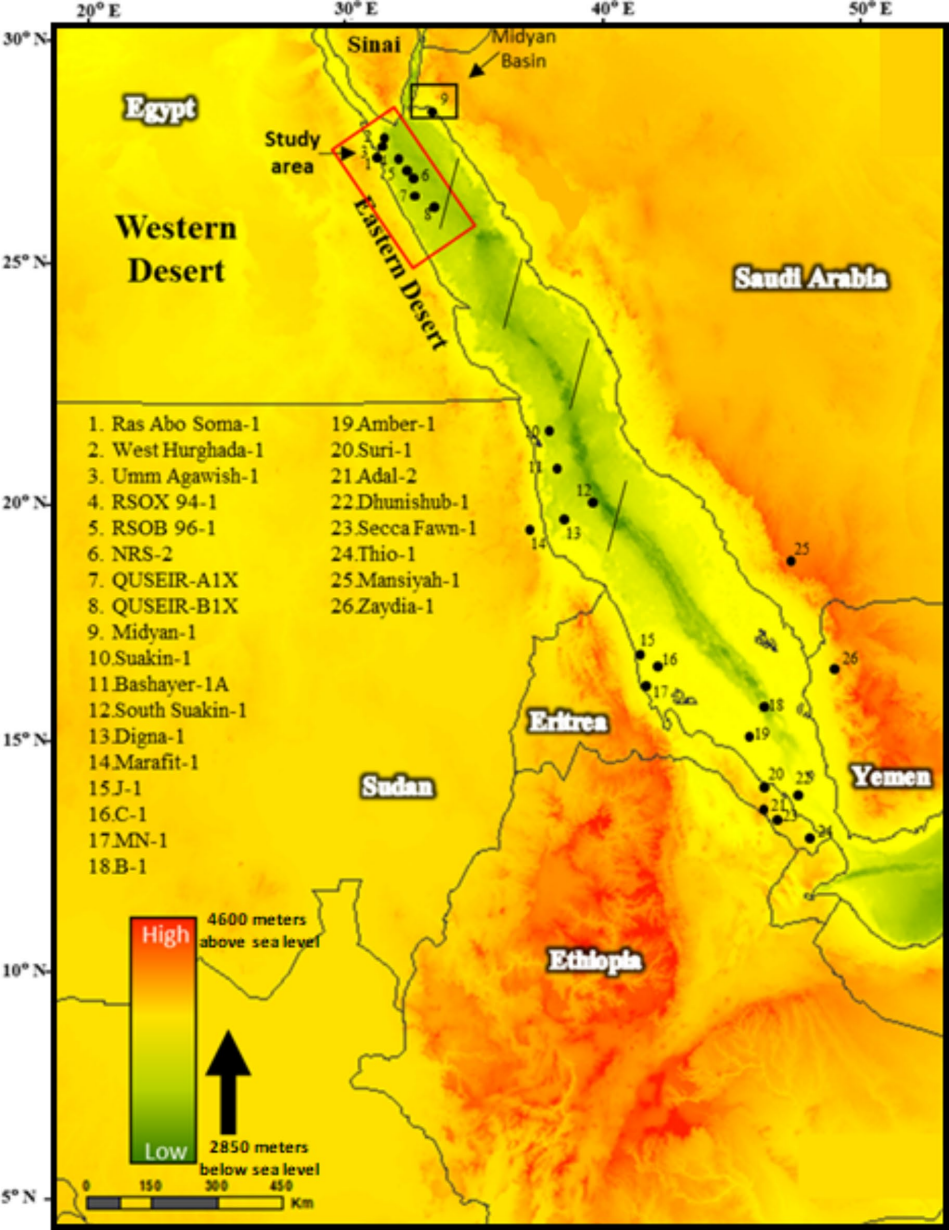
The Red Sea topography map showing most of the exploration history activities (Political boundaries may slightly vary)^43,67,8,60,6^. GEBCO’s 2023 model is used for the geographical projection^26^. Bathymetric extremes in the region, showing the lowest point in the Red Sea’s Central Trough at approximately 2,850 m below sea level, and Ethiopia’s highest elevation at Ras Dashen in the Simien Mountains, reaching around 4,550 m above sea level.

Focusing specifically on the Egyptian portion of the Red Sea, it is essential to delve into exploration history and hydrocarbon observations pertinent to this area. By examining publicly available information, we can trace the evolution of exploration activities and highlight key hydrocarbon discoveries relevant to Egypt^29,36^. This targeted approach provides insights into the petroleum potential within Egypt’s Red Sea region, enhancing our understanding of its specific geological context and exploration history. While regional exploration activities in Saudi Arabia, Eritrea, and other areas offer broader context, our primary focus remains on Egypt, ensuring that our analysis is directly applicable to the study area of interest (Fig. 3). This refined focus will contribute to a more detailed understanding of the petroleum potential in Egypt and align our study more closely with the central area of investigation.

### The Egyptian Red Sea

In 1978, the Ras Abou Soma-1 well, situated on the onshore side of the Egyptian Red Sea, successfully reached the pre-rift strata^2,3^. Drilling at a depth of 1279 m MD identified the basement, encountering a substantial 230 m of premium-quality Nubia reservoir^2,10^. Offshore wells such as the Quseir-B1x well^44,69^ along the Egyptian margin, reaching the basement, were likely drilled on the Precambrian basement block most up-dip. Offshore, on the hanging blocks where the pre-rift strata^69^ are less likely to be eroded or shifted by Red Sea hyperextension^44^, significant reservoirs and source rock intervals are likely preserved^41^. The Northern Egyptian Red Sea offshore wells, including RSOX 94 − 1, QUSEIR-A1X, and NRS-2, have shown indications of hydrocarbons as reported by^44,69^, and^2^. However, no pre-rift sediments were found. The presence of oil shows in nearby wells suggests that Cretaceous source rock units may be present within the Northern Egyptian Red Sea basin, enhancing the potential for oil discoveries in the stratigraphic sequence^22^.

### The Saudi Red Sea

Saudi Aramco focused on exploring undiscovered complex reservoirs on land and offshore in 2012. They discovered two gas fields, Shaur and Umm Ramil, and one oil field named Aslaf; Shaur being the first discovery on the Saudi offshore side^32,7^. Saudi Aramco also identified a natural gas reservoir in the Midyan basin, 25 km from the Red Sea town of Dhuba, with an initial production rate of about 10 MMCF/d of gas. The Al-Haryd oil field was discovered in 2013 as part of Saudi Aramco’s deep-water Red Sea frontier exploration program^52^. Additional oil accumulations were observed in the Midyan-1 well located in the Midyan basin^21^.

### The Eritrean Red Sea

In the Eritrean Red Sea region, signs of hydrocarbons were detected in wells J-1, MN-1, Thio-1, and B-1. Notably, the C-1 well experienced a gas blowout that lasted 55 days, originating from a depth of 3010 m. This information is documented by^4,67^, and^14^ (Fig. 3). Additionally, gas shows were identified in the Secca Fawn-1 well within a dolomitic layer intercalated with Amber salt. Promising oil indications and gas kicks were observed in wells Adal-2 and Suri-1 in the Dahlac Islands, as well as in Dhunishub-1 and Amber-1 wells within thin intra-evaporite black shale stringers in the extensive Amber Salt, as reported by^67^.

### The Sudanese Red Sea

The Lower to Upper Miocene Rudeis, Kareem, and Belayim Formations may have contained gas and oil source interbeds, but it is challenging to confirm this due to proximal penetration and their post-mature state in most analyzed wells^19^. The Sudanese Bashayer-1 A, South Suakin-1, Digna-1, Marafit-1, and Suakin-1 wells (Fig. 3) demonstrate a functioning petroleum system in the Sudanese Red Sea region, with oil sourced from the Miocene^29^. Condensate and gas discoveries at the Sudanese Red Sea, such as Suakin-1 and Bashayer-1 A, strongly support the existence of oil, likely sourced from the Rudeis, Kareem Formations, and intra-evaporite shales. Samples studied by Hadad and Abdullah^29^ observed TOC, HI, and hydrocarbon formation from kerogen pyrolysis, showing that the base Zeit source rocks are excellent for producing gas and condensate. Rudeis and Kareem Formations shales may have high-quality type-II kerogen compared to their analogs in Burqan and Maqna Group sources.

### The Yemeni Red Sea section

Pliocene shales from the post-rift Abbas Formation were collected and geochemically studied from an exploration well in the Tihamah Basin^31^. Preliminary assessments based on organic geochemical findings indicated that the Abbas shales have strong potential for petroleum production, with TOC contents > 1%. The majority of the Abbas shale samples had TOC contents of 1% and fair source potential. Hydrogen index values for the examined shales ranged from 96 to 234 mg HC/g TOC, indicating predominant type II-III kerogen. The Pliocene Abbas shales in the Tihamah Basin are relatively young and immature concerning the oil window and have not yet produced petroleum^31^. The Mansiyah-1 and Zaydia-1 wells in the Yemen region’s pre-salt package demonstrated substantial oil production (Fig. 3).

## Data and methodology

In this work, different data types and outcomes of numerous scientific research studies were gathered and incorporated for interpretation, such as well logs data sets, seismic, burial history, geochemical **and** geological outcrops (Gebel Duwi) to highlight the structural concept along with the transitional style between the Southern Gulf of Suez and Northern Egyptian Red Sea and delineate the potential plays^23,68,61,44,62,47,55,72,22,12,6^.

**Fig. 4:**
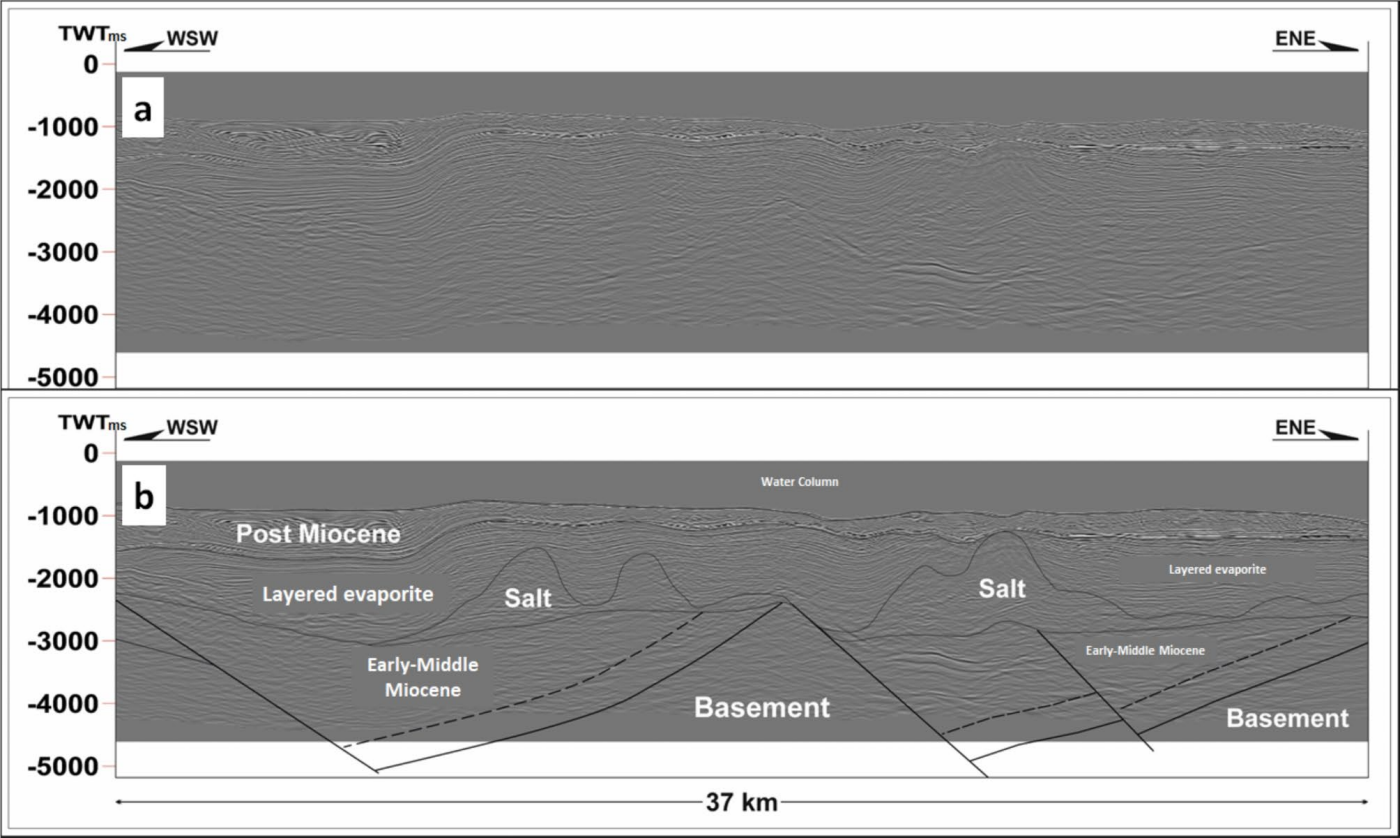
An interpreted seismic line (scale 1:1) illustrated the novel model that shows rotated fault blocks against the wedged syn-rift units above pre-rift and basement units, this model is used as an input to formulate Fig. 8 and part of Fig. 13b block diagram.

**Fig. 5:**
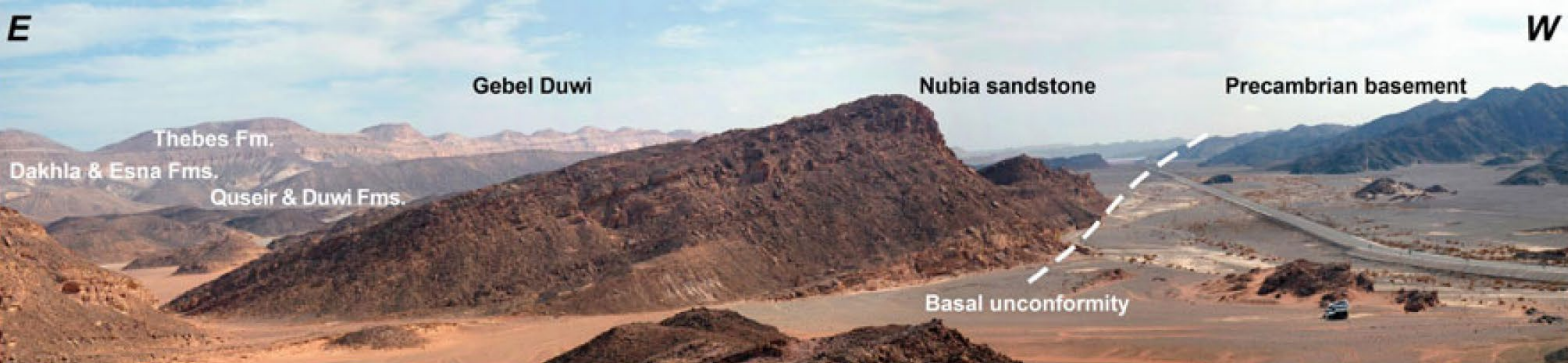
A view looking SE showing the pre-rift Cretaceous–Eocene section that serves as source and reservoir rock units that are well-preserved in the exposed rocks unconformably overlying basement in Gebel Duwi block (modified after^16^) to affirm Fig. 4 subsurface model.

Delivering precise and thorough evidence on the structural and stratigraphical aspects of the seismic prospect is the main goal of utilizing seismic interpretation^42,9,51,50,49^. Examples from the extracted seismic lines (Fig. 4) have been interpreted and linked to the Gebel Duwi outcrop (Figs. 5 and 13b), forming a regional scheme that sets the conceptual rifting structural style after integrating the other data types^16,43,72,12,6^. Moreover, a schematic cross-section (Fig. 11) aiming to correlate the Southern Gulf of Suez (Shab Ali area) with the RSOB 96 − 1 well area and highlight the subsidence of the Red Sea relative to the Gulf of Suez due to the Levant-Aqaba transform fault (after^43,72,65,22,69,12,6^) was performed.

## The petroleum system in the Northern Egyptian Red Sea

### Source rock

In the Northern Egyptian Red Sea region, the thickness and potential of source rocks vary. Miocene and pre-Miocene source rocks generally exhibit moderate to high potential but vary in maturity^1,3,5^. To clarify, we have applied geochemical techniques such as the Van Krevelen diagram and HI/Tmax plots to determine the type and quality of organic matter^90^. The organic matter in these units predominantly falls into Type II and mixed Type II/III categories, which are typically oil- and gas-prone. This classification is supported by the HI/Tmax diagrams, showing high hydrogen indices indicative of oil and gas potential^89,77^.

In contrast, the Gulf of Suez region shows more consistent and higher source rock potential due to its higher geothermal gradients, which enhance the maturation of source rocks^96,15^. Thicknesses in this region are more uniform, with significant source rock intervals observed^24^. The thickness range for various source rocks is characterized by Miocene source rocks typically ranging from 50 to 150 m, while pre-Miocene source rocks generally range from 70 to 200 m^16,17^.

#### Potential source rock formations

##### Belayim formation

The Belayim Formation exhibits promising source rock potential, with Total Organic Carbon (TOC) levels ranging from below 1.0% up to 4.0%. Pyrolysis yields (S2) typically range between 6.0 and 9.0 mg HC/g rock, with certain intervals showing even higher values. Thickness varies from 50 to 120 m^2,4^.

##### Kareem formation

The Kareem Formation also shows potential as a source rock, with TOC levels reaching up to 4.0% and pyrolysis yields generally between 6.0 and 9.0 mg HC/g rock. Its thickness ranges from 40 to 100 m^8,10^.

##### Rudeis formation

Comprising primarily marly shale, the Rudeis Formation has TOC values up to 4.0% and an average S2 value of 5.5 mg HC/g rock, indicating good source rock potential, especially in deeper basins. Thickness ranges from 60 to 130 m^6,12^.

##### Duwi, Sudr, and Matulla formations

These older formations exhibit significant source rock potential. Notably, the Matulla Formation has TOC readings exceeding 3.0% and S2 values above 6.0 mg HC/g rock. In the West Hurghada-1 well, TOC levels can reach as high as 12%, and S2 values up to 18 mg HC/g rock, with formation thickness ranging from 70 to 150 m^1,7^.

##### Hammam Faraun and Sidri components of the Belayim formation

These components show high hydrogen indices (> 300 mg HC/g TOC), suggesting significant oil and gas potential. Similarly, the Matulla Formation also displays high hydrogen indices, further supporting its hydrocarbon potential^14^.

#### Egyptian Red Sea source rocks insights from well RSO X94-1 internal reports

Reports from well RSO X94-1 (Fig. 9) offer further insights into thermal maturity in the region. Pyrolysis Tmax values for depths between 3300’ and 6870’ (Late to Middle Miocene) range from 405°C to 435°C, clustering between 418°C and 430°C, indicating immaturity for oil generation in these upper sections. Microscopic analysis of samples from 6900’ to 9340’ (Middle to Early Miocene) shows a maturity gradient, with the section remaining immature up to approximately 8000’, reaching early maturity between 8000’ and 9000’, and achieving middle maturity below 9000’. Increased spore color index and vitrinite reflectance data suggest high heat flux during the Early Miocene or fluid migration toward the basement as contributing factors in maturity progression.

#### RSO X94-1 well source rock evaluation

The assessment of various formations from internal data reveals the variability in source rock potential:

##### Pliocene formation (1386’–4525’)

Primarily sands interbedded with thin shale and evaporite layers. Organic carbon content and pyrolysis yields are low, with minor source potential detected at 3600’ and 3660’.

##### Zeit formation (Upper to Middle Miocene, 4525’–4720’)

Primarily sandstones and evaporites, showing very low organic content and no significant source potential.

##### South Gharib formation (4720’–6472’, Upper to Middle Miocene)

Comprising thin shale interbeds with high TOC and Type II kerogens. Despite the dominant halite lithology, which restricts source material, localized heavy, immature oil shows on the composite log indicate limited hydrocarbon generation potential.

##### Belayim formation (6472’–7042’, Middle Miocene) and Kareem Formation (7042’–7493’, Middle to Lower Miocene)

These formations exhibit limited yet notable source rock potential. Selective sampling indicates fair to good source capabilities, though their sandy nature restricts overall potential.

##### Rudeis formation (7493’–9340’, Lower Miocene)

Consisting mainly of shales and sandstones, this formation shows moderate pyrolysis yields and TOC values, indicating potential for heavier hydrocarbon contributions. Migrant hydrocarbon stains and oil shows in the South Gharib, Belayim, Kareem, and upper Rudeis Formations imply localized hydrocarbon migration, likely restricted to adjacent shale intervals.

**Fig. 6:**
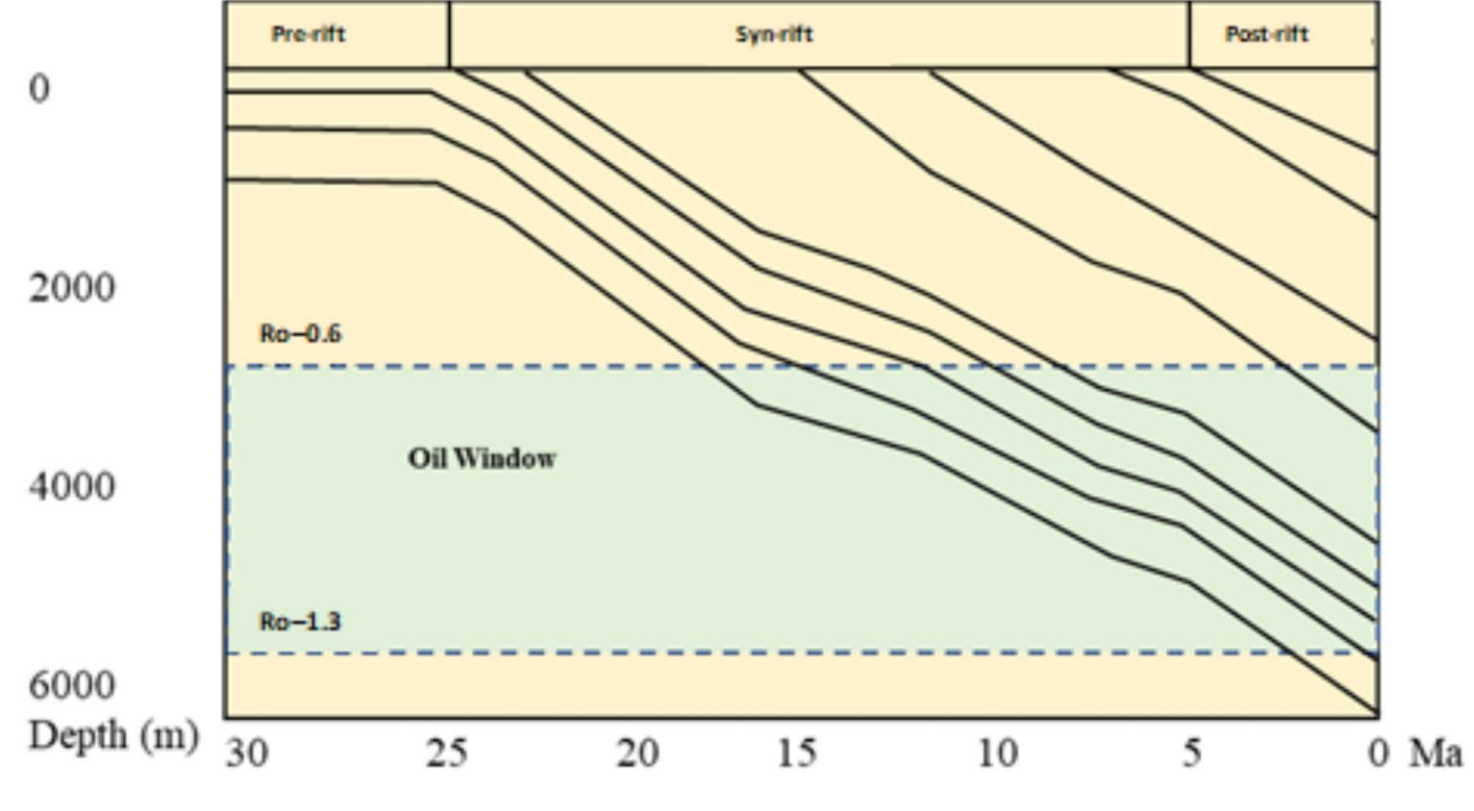
Burial history for miocene and pre-rift source rocks. (redrawn and modified after^33,21,62^)

In the Northern Egyptian Red Sea, the Oligocene period is marked by erosion or limited subsidence, affecting source rock development. Maturity levels generally range from mature to overmature stages, with Tmax, transformation ratio (TR), and vitrinite reflectance (Ro) employed to define oil windows, overmature zones, and gas generation areas across the region^26^. Burial history analyses of syn- and pre-rift source rocks provide a detailed understanding of source rock potential (Fig. 6)^21,33^.

**Fig. 7:**
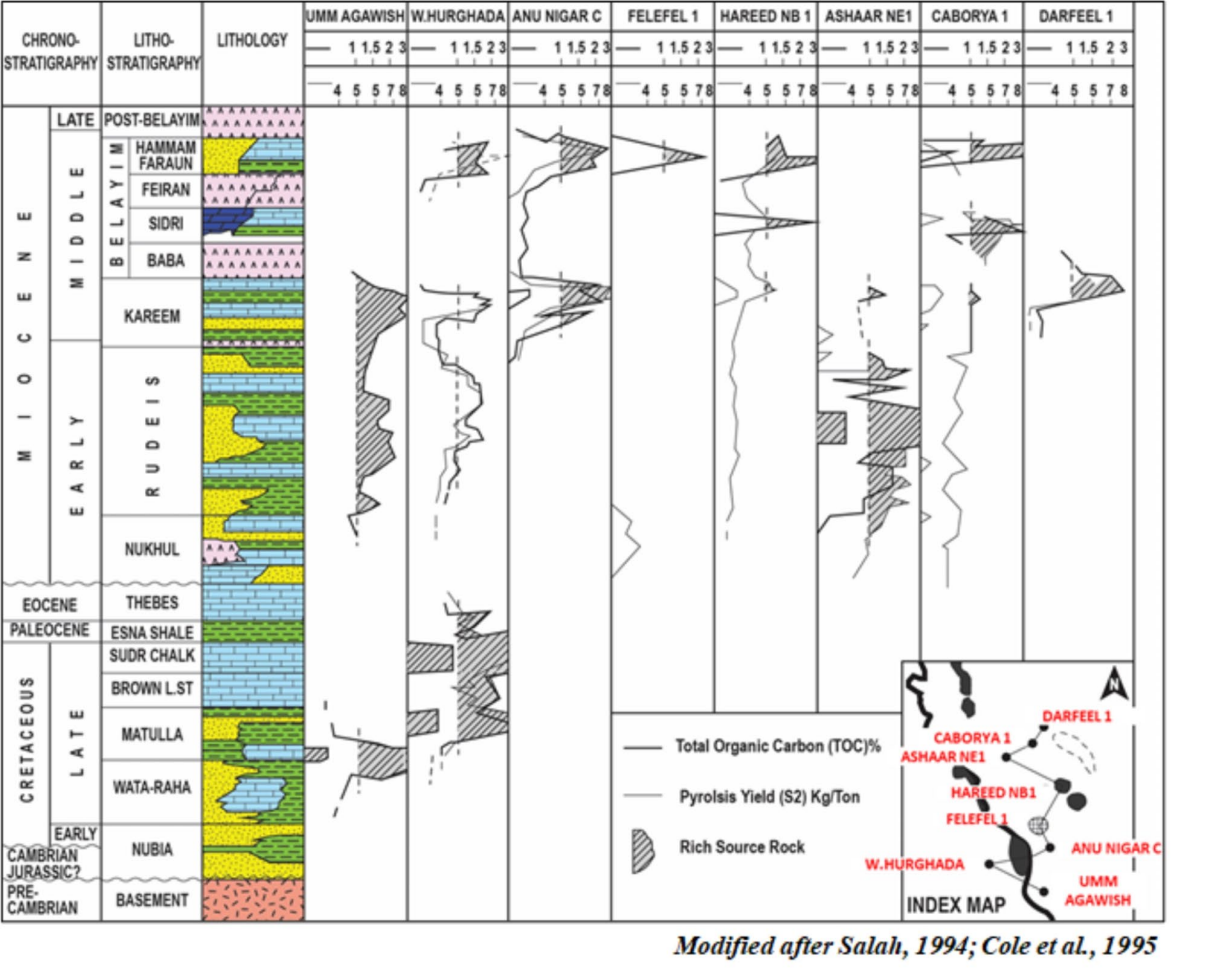
Source rock richness plot showing the total organic carbon content (%TOC) and the Rock-Eval pyrolysis yield (S2 in mgHC/g rock) of eight wells on the Southern Egyptian Gulf of Suez. (redrawn and modified after^62,21^).

Additional context is provided by source rock richness plots from nearby wells^21,62^ (Fig. 7), which show %TOC and Rock-Eval S2 yields across wells in the southern Egyptian Gulf of Suez, illustrating regional hydrocarbon richness and source rock potential.

### Northern Egyptian Red Sea potential reservoir rock units

In the North Egyptian Red Sea, three main types of reservoir rock units show promise, drawing from successful analogs in the Gulf of Suez and Saudi Arabia. Here’s a closer look at these potential targets:

#### Pre-rift Nubian reservoirs

Although we have seen Cretaceous rock units on the Saudi side, no exploration wells have yet encountered these pre-rift units offshore in Egypt^10,12,16^, and^40^. On land, Nubian sandstones are widespread along the Egyptian Red Sea coast and are also common in the Gulf of Suez^5^. These sandstones are known for their high beta values, which suggest they might only be found in isolated blocks that are elevated above basement lows throughout the basin. Even with these limitations, the potential of these pre-rift Nubian reservoirs remains significant and is not expected to diminish the overall potential of the Red Sea basin^3,4,9,16^ and^62^.

The Nubian sandstones, which originated in deltaic and fluvial environments, are characterized by coarse-grained, well-sorted deposits that are excellent for reservoir formations. These sandstones are anticipated to have high porosity and permeability, although confirming these petrophysical properties will require additional exploration^16,62^.

#### Syn-rift siliciclastic reservoirs

In the Miocene, the Gulf of Suez saw sedimentation patterns that closely mirror today’s drainage systems^44,71^. This period was crucial for forming large sedimentary basins, which are now prime targets for exploration. The thickest siliciclastic reservoirs from this syn-rift phase are likely to be found offshore, near ancient shorelines, and within rifted lows^52,67^.

Sediments from this period were transported from the Red Sea rift shoulders westward into the Nile catchment and then north into the Eastern Mediterranean^58,70^. Shaped by tectonic forces and sediment reworking, this process may have led to the formation of high-quality sandstones in some areas. Although early Miocene sandstones might show variable reservoir quality, with some being thin or poorly sorted^80,76^, regions with uplifted fault blocks could offer better reservoir quality due to the effects of sediment reworking^69,12,6^.

#### Syn-rift Carbonate reservoirs

Looking at carbonate reservoirs, we can draw parallels with the Midyan Basin in Saudi Arabia. During times of sea level rise, large benthic foraminifera and reef-building organisms like coralline red algae and scleractinian corals thrived, creating significant carbonate deposits. These carbonates often formed in high areas and might have seen improved reservoir quality due to exposure and karstification during periods of uplift or sea-level drop^40^.

These carbonates primarily formed as reefal deposits or on platform tops during high sea levels, which provided favorable conditions for reservoir development. The presence of vuggy porosity in these carbonates indicates they might have significant reservoir potential, though this is dependent on the extent of karstification and tectonic activity they experienced^40,43^.

### Trapping integrity, seals, and migration pathways

The Egyptian Red Sea basin is a region characterized by complex geological processes, and one of the prominent features influencing its structural evolution is salt tectonics. Salt structures play a crucial role in shaping the subsurface architecture of sedimentary basins, and the Red Sea is no exception to this phenomenon. The deposition and subsequent deformation of salt layers have profound implications for the basin’s structural development, impacting everything from the distribution of hydrocarbons to the overall tectonic framework.

In the Egyptian Red Sea context, extensive salt deposits contribute significantly to the basin’s structural complexity^26,44^. Over time, these ancient salt layers have undergone various phases of deformation, including movements like halokinesis, salt withdrawal, and the formation of salt diapirs. These processes have had a major impact on shaping the sedimentary layers above and play a crucial role in the region’s tectonic evolution^54,56^.

The Miocene anhydrite and salt are considered the regional ultimate seals in the Red Sea region. Also, it is anticipated that salt mobilization and stratigraphic pinch-out in the intra- and post-evaporite successions will create hydrocarbon traps. Play prediction and the definition of likely petroleum system components can benefit from applying analogs from nearby basins (e.g., the Gulf of Suez).

**Fig. 8:**
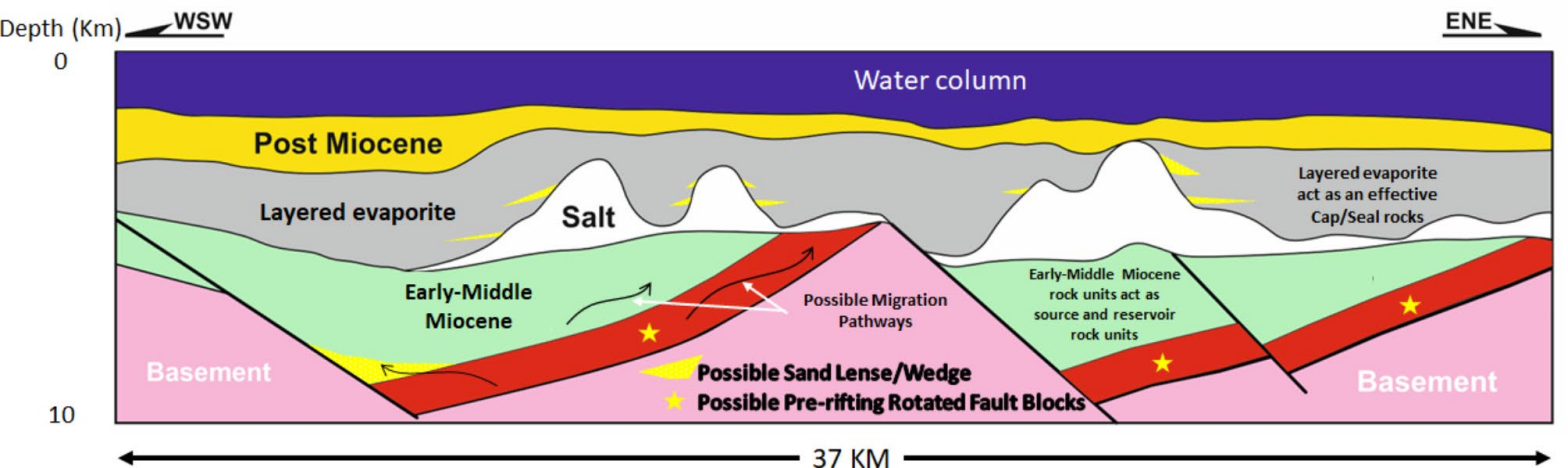
A new schematic cross-section (scale 1:1) illustrating the proposed play concept across the rotated basement blocks, based on the interpretation from the line shown in Fig. 4.

Figure 8 illustrates the Northern Egyptian Red Sea rotated block play concepts. Within the sub-Messinian salt stratigraphy, intra-formational, basinal mudstones immediately overlie proven reservoir horizons in the Lower Miocene stratigraphy of the Gulf of Suez (compiled from^43,72,6^).

Seals of a similar nature are anticipated across the Northern Red Sea, suggesting that the seal presence for lower syn-rift reservoirs poses a relatively low potential risk. Furthermore, a thick Upper Miocene evaporite provides an effective seal for Middle-Upper Miocene sub- and intra-salt reservoirs across much of the basin. These reservoirs are likely to be associated with structural traps, including pre-rift Nubian and syn-rift siliciclastic reservoirs, both typical of rift systems^71,72^ and^73^.

While the presence of Middle Miocene carbonate buildups beneath the salt is currently a hypothesis based on analogy with similar geological settings, identifying such targets would require validation through seismic data or outcrop evidence. In contrast, stratigraphic pinch-out traps are relatively easier to detect through well correlations, outcrop studies, or seismic interpretation, although they still depend on the presence of lateral and top seals. Salt mobilization-related traps and combined stratigraphic-structural traps are also expected, analogous to those found in the Southern Gulf of Suez^59,9^.

Finally, unconformities, faults, fractures, and permeable carrier beds developed along the Red Sea and Northern Egyptian Red Sea could provide migration pathways for hydrocarbons.

**Fig. 9:**
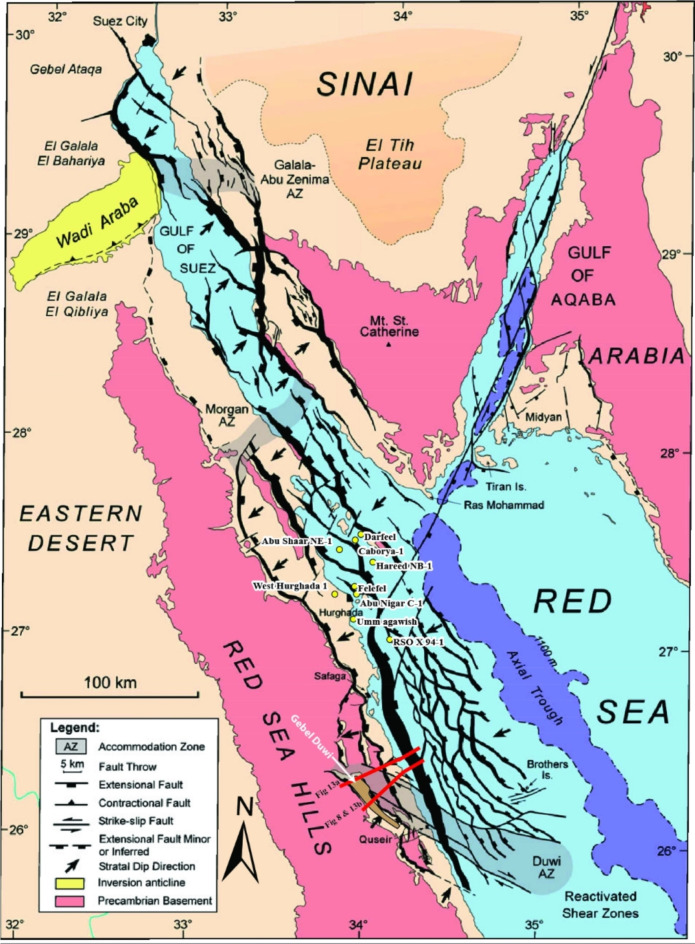
Main basement faults, with a width corresponding to the throw. (Modified from^16^). Plots of some wells related to Fig. 7 showing %TOC and Rock-Eval S2 yields for wells in the southern Egyptian Gulf of Suez. These figures offer key insights into the hydrocarbon richness and potential of source rocks against the structural architecture in the area.

## Results and discussion

### Northern Egyptian Red Sea and Southern Gulf of Suez correlation

The offshore Egyptian Red Sea side is distinguished by numerous fault trends that run perpendicular to the direction of the rift in the NW-SE trend, similar to the structural characteristics of the southern portion of the Gulf of Suez (Fig. 9)^17,20,22,18,6^. The area is characterized by a zigzag pattern of faulting that includes the Southern Gulf of Suez and Northern Egyptian Red Sea basement structuration^71,45,35,54^. Wells that were mentioned in Fig. 7 were plotted over the map presented in Fig. 9.

Numerous km-scale tilting blocks with substantial normal faults striking NW-SE have been described in the literature^16^, the Egyptian coast at and south of Quseir is dominated by a massive onshore half-graben formed by the west-dipping Gebel Duwi master fault that runs parallel to the beach, several asymmetrical salt domes and several visible salt walls offshore complicate the system, resulting in complex structural patterns, especially at depth^16^.

**Fig. 10:**
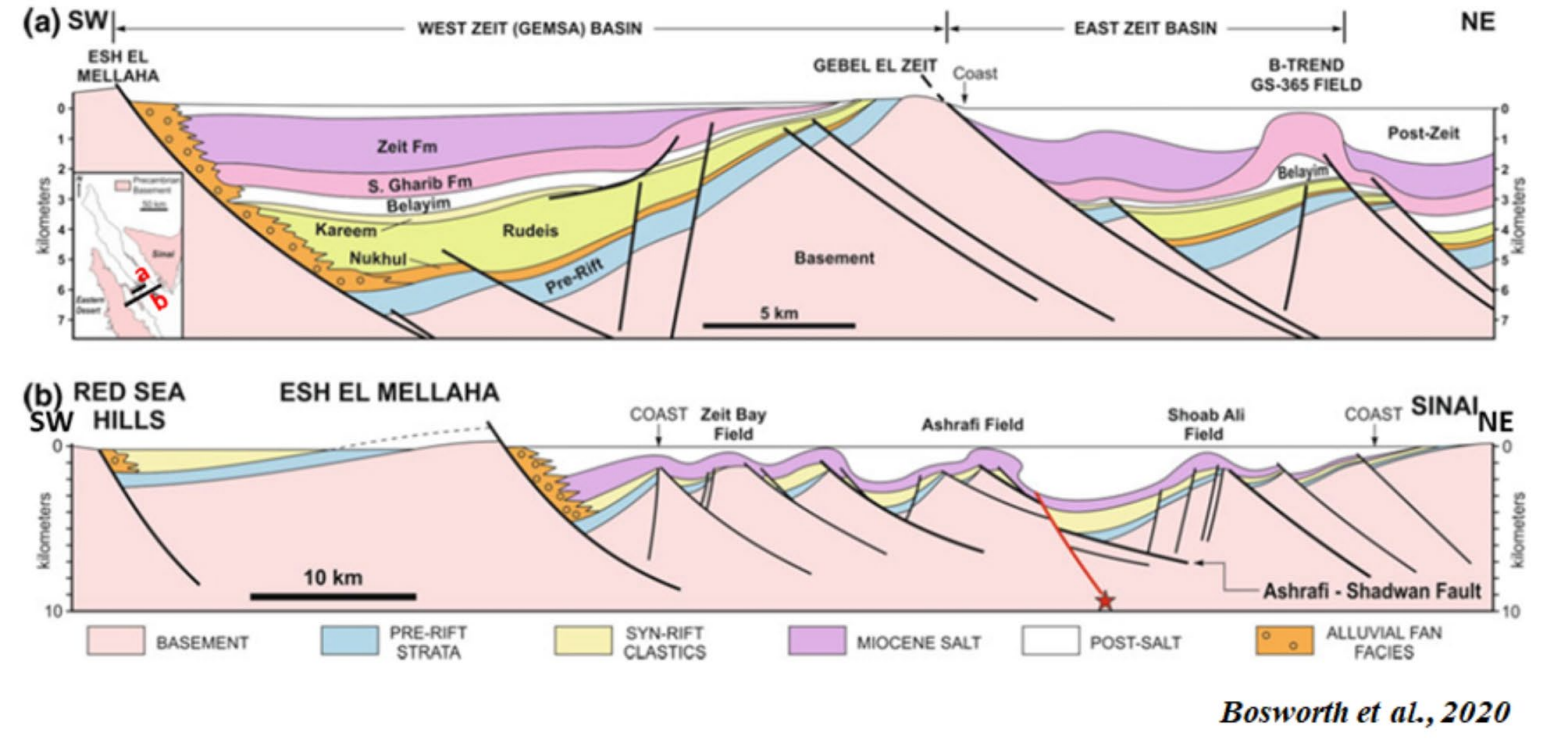
**(a)** A SW-NE regional scale structural cross-section/scheme of the East and West Zeit basins in the southern region of the Gulf of Suez. **(b)** An illustration of a SW-NE regional scale structural cross-section of Shoab Ali, Ashrafi, and Zeit Bay oil fields in the Southern Gulf of Suez shows rotational fault blocks up against wedge-shaped syn-rift units atop pre-rift and basement units (modified after^16^).

Regional scale structural cross-sections/schemes (Figs. 10a and b) across the Southern Gulf of Suez pass through Shoab Ali, Ashrafi, and Zeit Bay offshore oil fields, illustrating the rotated fault blocks against the wedged syn-rift units above pre-rift and basement units (after^16^). Block rotation and subsidence rates affect the distribution of the syn-rift units within these wedge-shaped packages. The earliest syn-rift units are in the downdip parts of these basins, close to the major fault that encircles these blocks. The maximum thickness of the wedge formations discovered in the syn-rift rocks adjacent to the enclosing faults is shown in Fig. 10a and b. The West Zeit and East Zeit Basins in the southwest corner of the Suez have wedge-shaped syn-pre-rift unit geometries similar to the Western Desert in northern Egypt^50^.

**Fig. 11:**
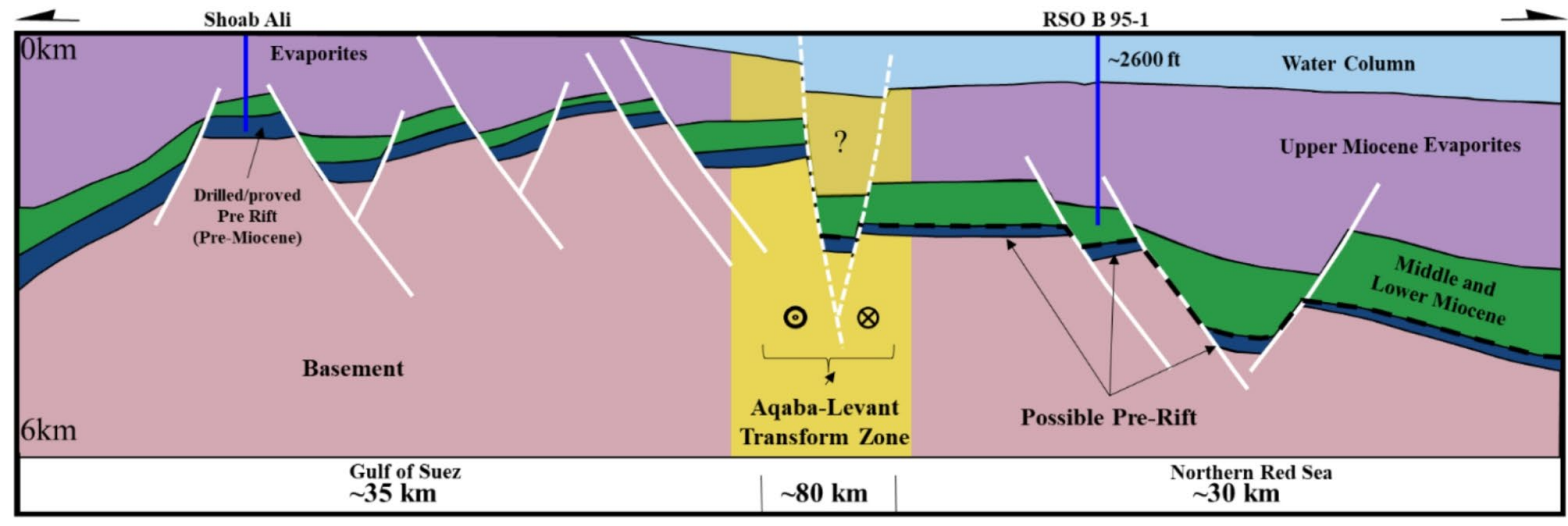
Schematic Regional NW-SE trending cross-section (∼ 140 km) tie between the Gulf of Suez and North Egyptian Red Sea, (compiled after;^16,37,20,65,39,66,5,22,18,69,12,6^).

A regional schematic NW-SE trending profile of about (∼ 140 km) length (Fig. 11) tie between the Gulf of Suez and the Northern Egyptian Red Sea shows that the alteration of the basement relief due to the Levant-Aqaba transform fault^39,66^ that splits the Southern Gulf of Suez and the North Egyptian Red Sea^16^, and both have southwest strata dipping that resembles the Northern Gulf of Suez (compiled after^20,65,5,22,18,69,12,6,38,48^), which would expose optimistic plays in the Northern Egyptian Red Sea like the Southern Gulf of Suez petroliferous basin that has recorded many hydrocarbon discoveries in the last decades. This original research article also presented a couple of block diagrams to examine the controlling conceptual structure; these two block diagrams run NE–SW perpendicular to the main NW-SE structural elements in the region. The outcrop cross-sections by Bosworth et al.^16^ for Gebel Duwi outcrops and the corresponding offshore seismic lines of Azab et al.^12^ were linked together to show the stratigraphical correlations crucial for the petroleum industry. .

### Northern Egyptian Red Sea surface-to-subsurface correlations and block diagram

**Fig. 12:**
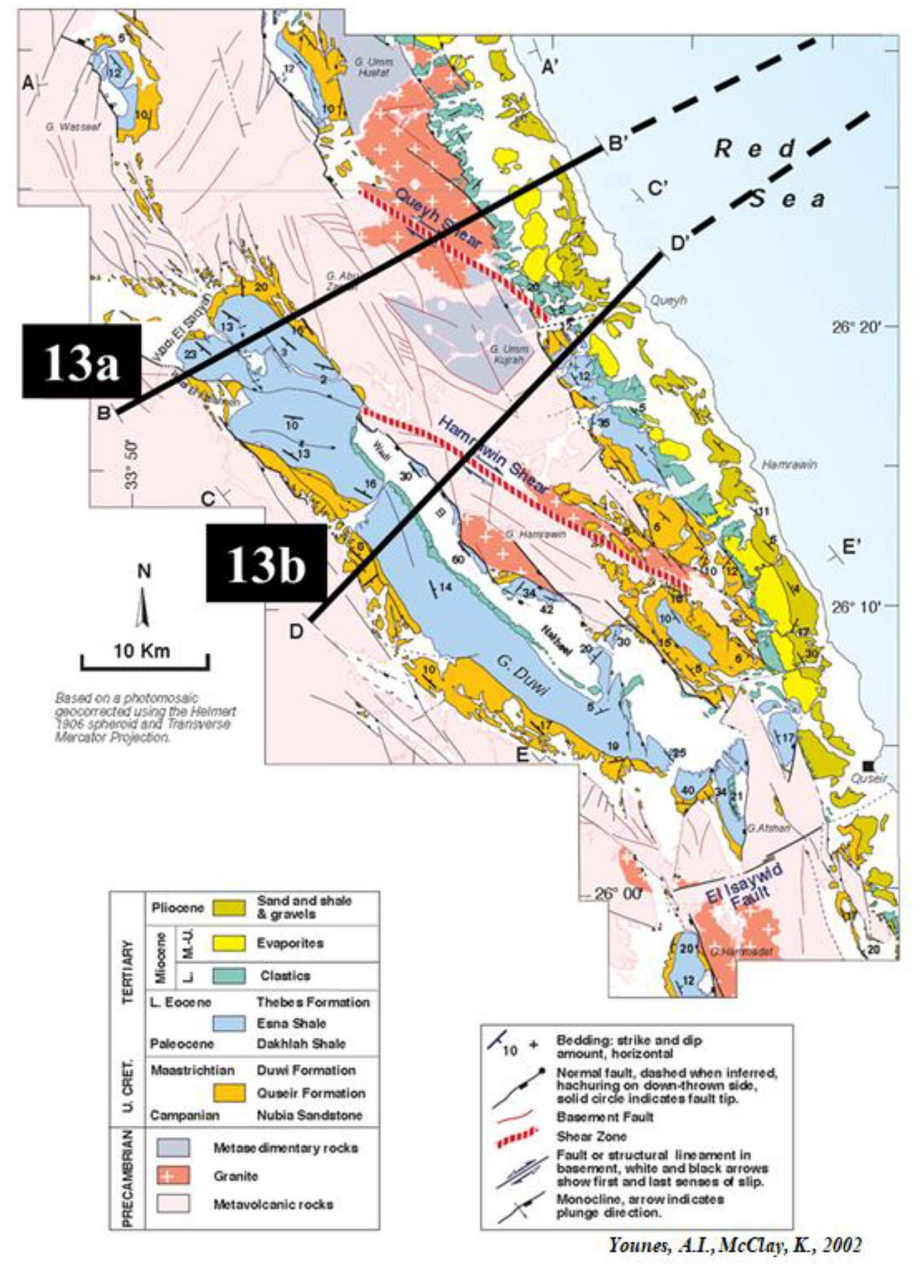
Geologic map of the Duwi area showing the location map of the Onshore to offshore schemes on the Egyptian Red Sea (Modified after^72^).

**Fig. 13:**
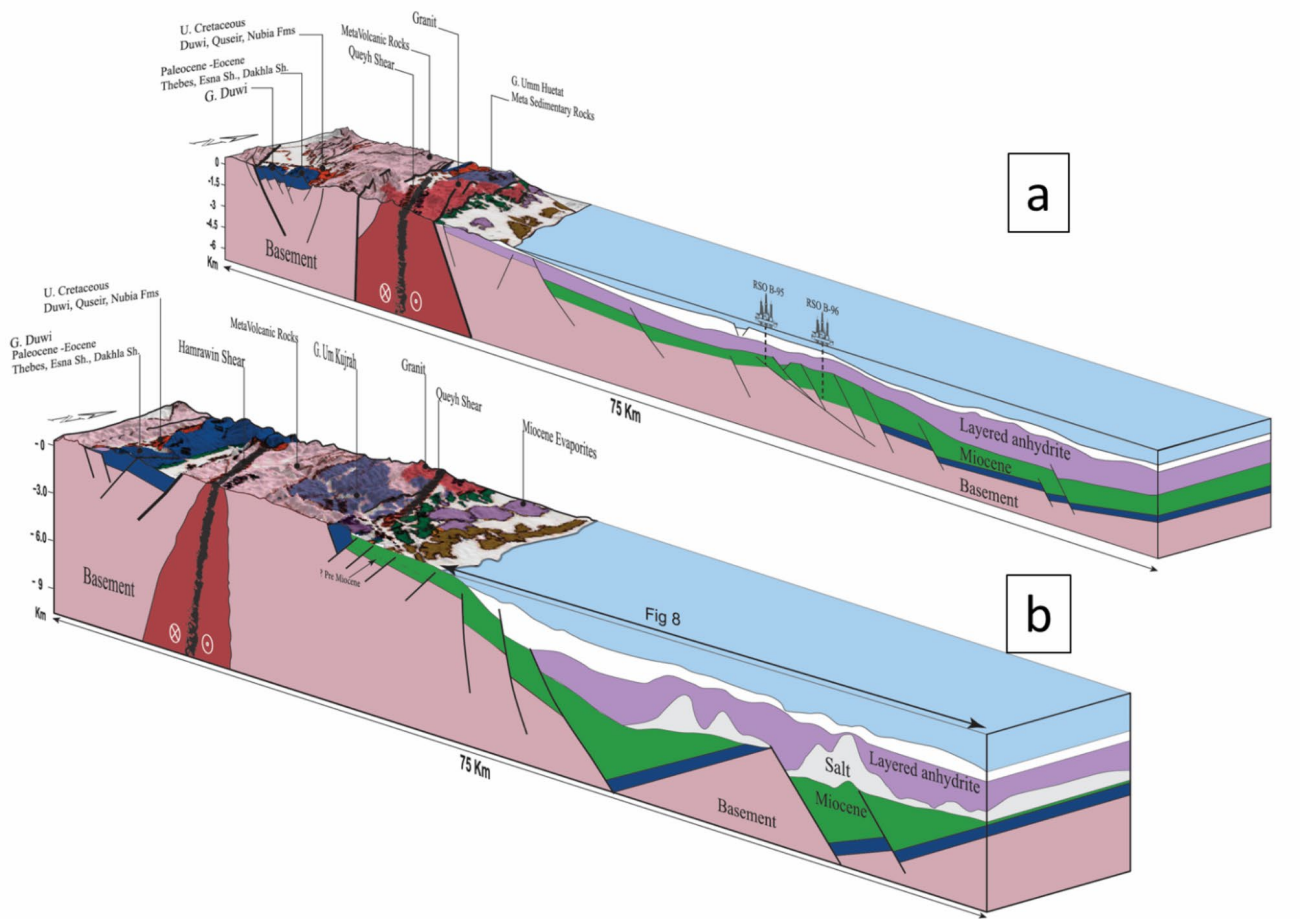
Two Geological block diagrams (scale 1:1) encapsulating the primary findings and insights on the Northern Egyptian Red Sea, this complicated geological block diagram, meticulously compiled by integrating information from both Figs. 4, 12, and 13, involvedly correlates the surface and subsurface geology. Surface geology annotations from^72^.

Figure 12 shows a detailed field geological map of the Duwi area and the location map of the onshore and offshore schemes on the Egyptian Red Sea^72^. On the Egyptian Offshore Red Sea side, the geological analysis around well Quseir-B1X has uncovered differences between the initially proposed structural model and the actual one. The initial model, based on a straightforward horst interpretation, anticipated thick pre-rift strata within the upthrown horst structure. However, upon drilling and examination of the up-dip basement beneath upper Miocene strata, a new model emerged (Fig. 13b). This innovative model reveals a distinct wedging shape, suggesting the presence of substantial sediment thickness in the down-dip direction, contrasting with earlier expectations. These insights were derived from signal enhancements on seismic sections using seismic attributes, leading to a more refined understanding of the subsurface. The legacy cross-section running WSW-ENE across the well site further supports these findings.

This study’s results align with findings from Stockli and Bosworth^70^, suggesting that the sediments dip in the offshore Northern Egyptian Red Sea is probably comparable to the regimes observed in both the Northern and Southern Gulf of Suez. The study pointed out that the highest thickness of the wedge-shaped sediments in the Southern Gulf of Suez region and Northern Egyptian Red Sea is found near the bounding faults, and massive normal faults surround the half-graben basins where they were deposited. Our study presumes that these units may pinch out and wedge out in the up-dip direction, and patches of thick sedimentary sections have been traced to show possible NW basins.

### Surface-to-subsurface correlations in the Northern Egyptian Red Sea

(Figs. 13a and b) were developed by compiling outcrops, and the corresponding offshore seismic data. Geological block diagrams in Fig. 13 serve as the focal point of this research article, encapsulating the primary findings and insights derived from studying the previous work on the Northern Egyptian Red Sea. This complicated geological block diagram, meticulously compiled by integrating information from both Figs. 12 and 13, involvedly correlates the surface and subsurface geology. The illustration offers a comprehensive depiction of the geological structures, and stratigraphy, providing a visual synthesis of the intricate interplay between surface topography and subsurface geological formations in the Northern Egyptian Red Sea.

Figure 13 enhances the understanding of the geological complexities inherent in this region, contributing significantly to the broader scientific discourse on the geological dynamics of the Red Sea area. Patches of thick sedimentary sections have been traced showing possible NW basins that tend to be 7–10 km thick. The compiled diagrams suggest that the pre-rift strata exist in the subsurface succession on the offshore Northern Egyptian Red Sea. Blocks tilting affects how the sediments are distributed in the shape of wedged packages. The Northern Egyptian Red Sea displays wedge-shaped pre- and syn-rift (sediments) unit geometries, and significant reserves are expected to be discovered in the thick sedimentary sections within the down-dip directions of the rotated faulted blocks.

## Conclusions

- The Red Sea remains largely under-explored, with the Northern Egyptian Red Sea, particularly in needing further investigation due to limited borehole data, sparse case studies, and inadequate seismic information.
- The research integrated geological block diagrams and regional schematic cross-sections, combining field geology of the onshore Gebel Duwi area and offshore subsurface geology, to connect the Southern Gulf of Suez with the Northern Egyptian Red Sea offshore wells.
- Syn- and pre-rift organic-rich source units in the Northern Egyptian Red Sea could potentially generate oil and gas, similar to the Southern Gulf of Suez, indicating possible capped reservoirs in these units.
- Both the Southern Gulf of Suez and the Northern Egyptian Red Sea are influenced by the Levant-Aqaba transform fault, leading to southwest-dipping strata and a structural style similar to the Northern Gulf of Suez.
- Rotated basement faulted blocks, extending from the shoreline to the axial trough due to rifting, exhibit wedged Miocene sections. Most offshore boreholes reached Precambrian rocks directly below the Upper Miocene.
- The research suggests that many offshore wells may have been drilled off-structure, capturing up-dip basement sections. Similar successful drilling strategies in the Gulf of Suez, such as in the Ashrafi area, could inform future exploration.
- The study’s findings could stimulate further hydrocarbon exploration and development in the Northern Egyptian Red Sea region.

## Data Availability

Data are however available from the author (Mr. Mustafa Hassan) upon reasonable and with permission of [ The Egyptian General Petroleum Cooperation].
